# Distribution of single‐nucleotide variants on protein–protein interaction sites and its relationship with minor allele frequency

**DOI:** 10.1002/pro.2845

**Published:** 2015-12-09

**Authors:** Hafumi Nishi, Junichi Nakata, Kengo Kinoshita

**Affiliations:** ^1^ Department of Applied Information Sciences, Graduate School of Information SciencesTohoku UniversityMiyagi980‐8597Japan; ^2^ Tohoku Medical Megabank Organization, Tohoku UniversityMiyagi980‐8573Japan; ^3^ Institute of Development, Aging, and Cancer, Tohoku UniversityMiyagi980‐8575Japan

**Keywords:** protein complex, protein–protein interface, rare variants, nonsynonymous mutations, 3D structure

## Abstract

Recent advances in DNA sequencing techniques have identified rare single‐nucleotide variants with less than 1% minor allele frequency. Despite the growing interest and physiological importance of rare variants in genome sciences, less attention has been paid to the allele frequency of variants in protein sciences. To elucidate the characteristics of genetic variants on protein interaction sites, from the viewpoints of the allele frequency and the structural position of variants, we mapped about 20,000 human SNVs onto protein complexes. We found that variants are less abundant in protein interfaces, and specifically the core regions of interfaces. The tendency to “avoid” the interfacial core is stronger among common variants than rare variants. As amino acid substitutions, the trend of mutating amino acids among rare variants is consistent in different interfacial regions, reflecting the fact that rare variants result from random mutations in DNA sequences, whereas amino acid changes of common variants vary between the interfacial core and rim regions, possibly due to functional constraints on proteins. This study illustrated how the allele frequency of variants relates to the protein structural regions and the functional sites in general and will lead to deeper understanding of the potential deleteriousness of rare variants at the structural level. Exceptional cases of the observed trends will shed light on the limitations of structural approaches to evaluate the functional impacts of variants.

## Introduction

Single‐nucleotide variants (SNVs) are nucleotide differences in DNA sequences among individual genomes, which may cause phenotypic variations and potentially some diseases. Genome‐wide association studies (GWAS) have successfully identified disease‐related variants in a statistical manner, and some were subsequently verified as disease‐causing variants by biochemical and cell biological experiments.^1^, ^2^ Furthermore, recent advances in DNA sequencing techniques now allow large‐scale genome analyses, which can identify rare variants with less than 1% minor allele frequency (MAF).^3^, ^4^ According to the vast accumulation of genomic data, variants with high MAFs (common variants) are not considered to cause severe effects, and rare variants will often have larger impacts for diseases than common variants.^5^ GWAS analyses are based on the statistical association between genomic differences and phenotypic changes, and thus, they are not effective for rare genomic variations, unless a vast number of samples are available.

To overcome this difficulty, numerous computational studies have been performed on nonsynonymous variants from structural viewpoints to evaluate the impact of individual mutations on protein structures and functions in a physicochemical manner.^6^, ^7^ The structural studies suggested that disease‐causing variants can affect the stabilities, dynamics, and interactions of proteins.^8^, ^9^, ^10^ For example, de Beer *et al*.^11^ examined nonsynonymous variants in 1000 genome data and described the differences between the neutral variants and the disease‐causing mutations in terms of the substitution patterns and the secondary structures. Recently, David and Sternberg^12^ conducted a comparison between deleterious mutations and less harmful polymorphisms on protein interfaces and showed that less harmful mutations are more abundant on the rims of interfaces when compared with the interfacial core regions. Similarly, Lu *et al*.^13^ observed that polymorphisms are overrepresented in disordered regions, whereas disease‐causing mutations are underrepresented. All of these studies promoted the understanding of the functional and/or structural impacts of variations; however, very little attention has been paid to the influence of the allele frequency of variants on protein structures despite the growing awareness of the functional consequences of rare variants.

In this study, we tried to clarify the general characteristics of SNVs on protein interactions, by specifically focusing on the relationship between MAF and the protein interfacial location of variants. We collected nonsynonymous SNV data from the NHLBI Exome Sequencing Project^14^ and mapped them onto protein complex structures. Each variant was then classified into different categories, based on its MAF (rare, intermediate, and common) and its position on the interface (interfacial core, rim, and support). Finally, we observed the relationship between DNA‐level random mutation patterns and biased patterns at the protein level.

## Results and Discussion

### Interfacial location and minor allele frequency of SNVs

We found 20,305 variants on 1,343 protein complexes by mapping all of the variants in the NHLBI Exome Sequencing Project^14^ onto protein complex structures via the RefSeq^15^ protein sequences by using BLAST,^16^ where the protein complexes were obtained from PDBePISA.^17^

First, we checked the location of variants with different MAFs in the interfaces. The definition of interfacial location was taken from the study of Levy,^18^ which classified interfacial sites into three categories: core, rim, and support, based on the relative accessible surface area (rASA) of the amino acid residue at each site, and its change on binding (ΔrASA). All of the interfacial sites are identified as the residues with ΔrASA > 0. Among them, the support sites are considered to be buried (rASA < 0.25) even in the monomer state, whereas the rim sites are defined as being exposed (rASA > 0.25) in the complex state. In a similar manner, the core sites are exposed in the monomer states and become buried on binding. According to these definitions, residues in the core sites are more likely to contribute to protein binding than rim or support residues, whereas variations on support sites will be more responsible for protein stability than protein–protein interactions. As shown in Table 1, the ratios of variants in the core, rim, and support sites were 37%, 43%, and 20% (1550, 1813, and 841 sites) and those in the nonvariant sites were 38%, 37%, and 25% (27,154, 26,799, and 17,725 sites), respectively. The differences in the ratios revealed the weak trend that the occurrences of variants are suppressed at crucial sites for protein structure (support sites) and binding (core sites), where the *P*‐value of the distribution difference is 2.4 × 10^−16^ by the *χ* ^2^ test.

**Table 1:** Distributions of Single‐Nucleotide Variants on Proteins

	Interface				Non‐interface		Total
	Core	Support	Rim	Total	Protein Internal	Protein Surface	
All variants	1550	841	1813	4204	7207	8894	20,305
Rare	1516	828	1748	4092	7015	8533	19,640
Intermediate	20	8	29	57	109	176	342
Common	14	5	36	55	83	185	323
Non‐variant	27,154	17,725	26,799	71,678	146,816	128,860	347,354

To assess the effect of MAF on this trend, we divided the distribution according to the MAF values and observed that this trend became stronger for common variants. More precisely, the ratio of common variants on rim sites was 67% (36 of 55 sites), which was significantly higher than that of rare variants (*P*‐value: 0.003 by the *χ* ^2^ test between the ratios of rare and common variants on interfaces).

The trends of MAF of variants on different interfacial regions were also examined. The ratios of rare, intermediate, and common variants on interfaces were 97.3%, 1.4%, and 1.3% (4092, 57, and 55 sites), respectively (Table 1). The ratio of nonrare variants (intermediate or common) on noninterfacial protein surfaces was 4.0% (176 + 185) and that of protein interiors was 2.7% (109 + 83), indicating that nonrare variants slightly tend to be suppressed at protein interfaces (2.7%; intermediate: 1.4% and common: 1.3%) and are similar to protein interiors (*P*‐value: 0.0003 by *χ* ^2^ test between interfaces and surfaces).

When the interface residues were divided into the three categories, the core and support regions were found to have a protein interior‐like trend, where nonrare variants were suppressed (2%), whereas the trend of the rim regions was similar to the protein surfaces (nonrare variant ratio: 3.6%). In short, SNVs have different preferences for interfacial locations depending on their MAFs, namely, nonrare variants “avoid” interfacial core and support regions when compared with rim regions and vice versa. This bias seems to be reasonable and coincides with the previous report,^12^ because variants should not be too harmful to spread in a population. A mutation on the interfacial core or support regions will cause the dissociation of functional oligomers, which could abolish the biological functions of proteins.

### Residue substitutions of rare and nonrare variants on interfaces

To elucidate the detailed features of the rare and nonrare variants on interfaces, we examined the mutating (before mutation) and mutated (after mutation) amino acids in the interfacial and MAF categories. Because of the similar distributions in the core and support regions, we combined them for the statistics. Based on the raw counts of amino acid substitutions (Supporting Information Fig. S1), arginine is an outlier in both the rim and core and support regions, and it mutates about four times more frequently than the second mutating amino acids among the rare variants in rim regions. This is consistent with the previous report^11^ and is possibly a result of CG suppression. Four of the six arginine codons contain CG, and thus, arginine residues are expected to be more frequently mutated to other residues. Several other amino acids showed different characteristics between the rim and core and support regions. For example, in the core and support regions, mutations to valine are more frequently observed when compared with other residues, whereas this tendency was not observed in the rim regions.

**Figure 1:**
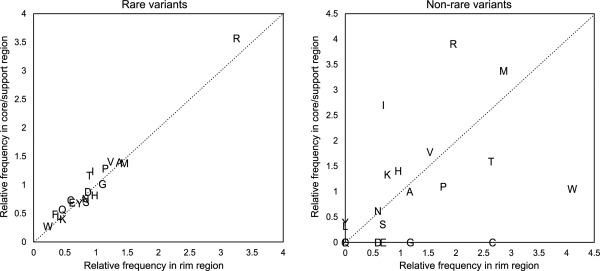
Relative frequency of each amino acid among rare and nonrare variants. Left: Rare variants; right: nonrare (intermediate and common) variants. The relative frequency of an amino acid is defined as the ratio of its percentages among variant and nonvariant sites. In nonrare variants, Q and F are overlapped at (0,0).

The raw counts provided some insights into the differences in the mutation patterns in the interfacial regions, as shown above. However, the raw counts must be normalized with the amino acid compositions to compare the amino acid changes due to the MAF differences in interfacial regions, because various interfacial regions have different amino acid compositions (Supporting Information Fig. S2). For example, rim regions contain more arginine residues when compared with core and support regions.^18^ For the normalization, we defined the relative frequencies of mutating residues as the ratio of the percentages of amino acids in variant sites and nonvariant sites. For example, the relative frequency of arginine among rare variants and rim regions is calculated as 28.2/8.67 = 3.25, where the mutating rate of arginine in rare variants is 28.2% and that in nonvariant sites is 8.67%. As a result (Fig. 1), we found that the relative frequencies of the rim and core/support regions for rare variants correlated well with each other (0.98 overall correlation coefficient; 0.93 without Arg); however, the correlation completely disappeared among the intermediate/common variants. The high correlation in the rare variants means that there are no differences in the mutating amino acids between the rim and core/support regions among the rare variants, indicating that rare mutations occur on protein interfaces randomly. In contrast, the weak correlation for nonrare variants implies the existence of some preferences for amino acid substitutions at different interfacial locations. This seems quite reasonable, because common variants were selected and spread in a population as nonharmful mutations during the course of evolution, but rare variants are new, and even unfavorable mutations may not be eliminated from the population by selections. Mutations in DNA sequences occur randomly, and rare variants reflect the randomness of mutations at the DNA level, whereas the bias of amino acids in common variants may reflect the functional constraints at the protein level.

Note that we did not perform the relative frequency analyses on the mutated residues, because these analyses are not as simple as those on the mutating residues. The analyses require not only the interfacial amino acid compositions but also the limited amino acid substitution patterns generated by single‐nucleotide changes in the DNA codon table and the frequency of each substitution pattern estimated by all SNVs in a population. In other words, the numbers of mutated residues are complicatedly biased by these factors; however, we believe that after appropriate normalization, the mutated residues will also show similar characteristics to the mutating residues: the patterns of rare variants result from random mutations, whereas those of common variants result from functional constraints.

We also checked the ratio of transition and transversion on nucleotide substitutions and found that they were almost the same (about 71% vs. 29%) among different MAFs and interfacial categories (*P*‐values of *χ* ^2^ tests: 0.74 for MAF and 0.73 for interfacial categories). On the other hand, when we focused on some specific residue substitutions, such as within β‐branched (Ile and Val, attained by transition) and between β‐branched and non‐β‐branched hydrophobic residues (Ile and Val from/to Leu, which require transversion), the transition/transversion ratios were different among nonrare and rare variants (*P*‐value: 0.010 by Fisher's exact test), namely, nonrare variants included more substitutions between Ile and Val (79% for nonrare and 65% for rare variants). As substitutions within β‐branched amino acids are known to be less harmful than those between β‐branched and non‐β‐branched, this trend is also considered as an example for protein‐level constraint on common variants.

### An exceptional case and a possible limitation of structural analyses

Although most SNVs were rare and tended to exist on rim regions, as shown above, 19 common variants were unexpectedly found in the core or support regions of interfaces. By inspecting these variants, we confirmed that most of the variants were substitutions between amino acids with similar sizes and physicochemical properties, such as threonine to methionine. However, the R287Q variant (MAF = 10.2%) in bifunctional epoxide hydrolase 2 (EPHX2) is an exception among these common variants.

**Figure 2:**
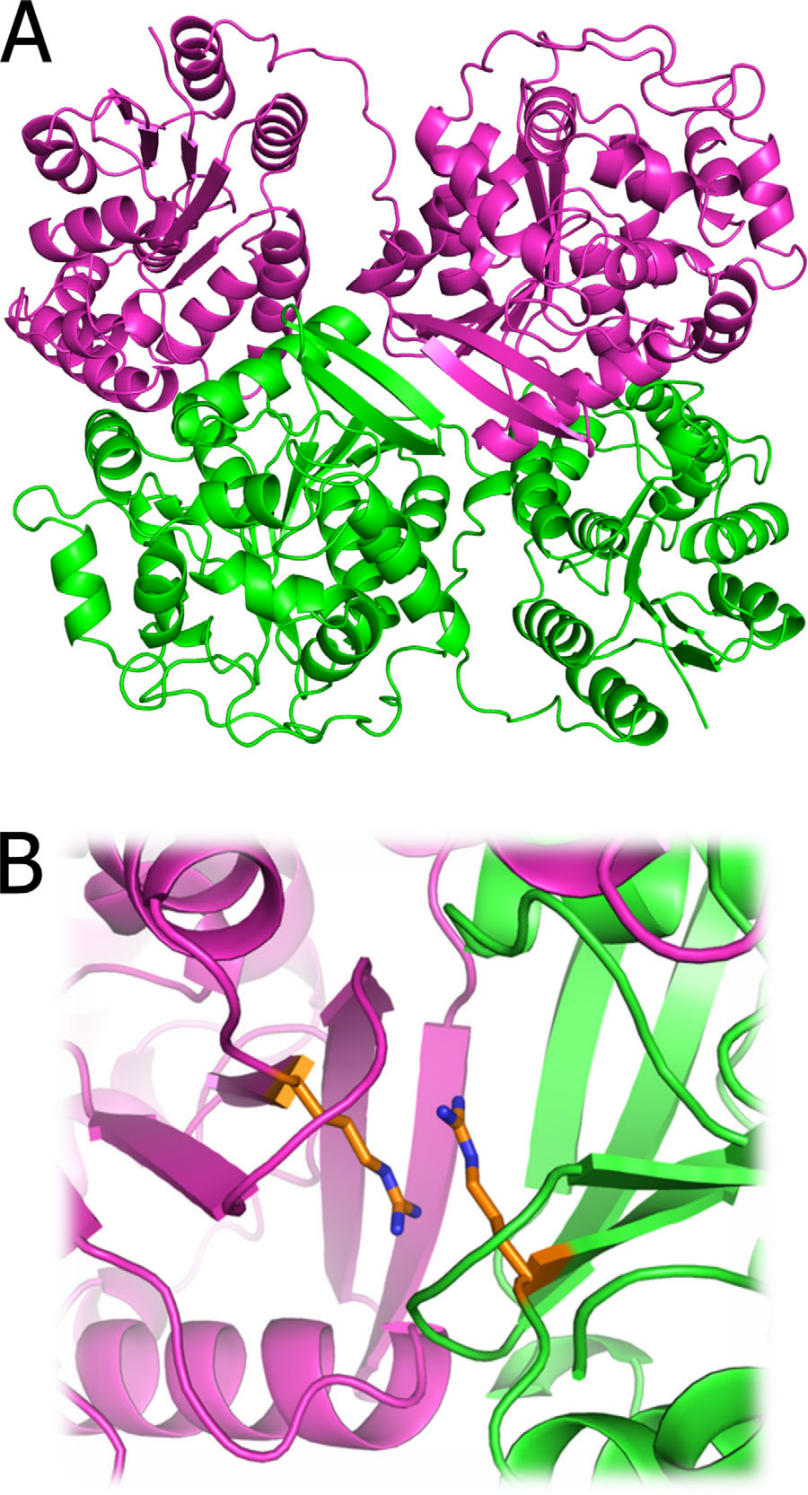
An example of common variants on a protein interface core. A: The dimeric structure of epoxide hydrolase (PDB ID: 4j03). B: The variant site (Arg287) on the dimeric interface. The mutating arginine residues are shown as orange stick models. The figure was prepared with PyMol.^19^

EPHX2 forms a homodimer *in vivo*, and dimer formation is required for its biological activity [Fig. 2(A)].^20^ As the Arg287 residue exists on the interface core region (ΔrASA: 0.25 Å^2^; ΔASA: 60.49 Å^2^) and interacts with the identical residue on the other subunit, the mutation seemed to have a large impact on its binding and functional activity [Fig. 2(B)]. Indeed, biochemical assays *in vitro* revealed that the R287Q mutation affects the binding constant of the dimer. Its dissociation constant changed from 5 pM (wild type) to 90 pM (R287Q), indicating a possible functional problem caused by this mutation. However, from the viewpoint of a high MAF, this mutation may not be harmful. At a glance, the biochemical assay and the high MAF are contradictory; however, the change in the dissociation constant caused by this mutation is compensated by the high concentration of the protein in tissues.^20^ Although the concentrations differ among various tissues, the concentration of this protein is at least 3 nM, which is sufficient for dimer formation. This example illustrates that critical substitutions from the viewpoint of protein structures may not always be harmful from the viewpoint of their functions. Therefore, other environmental conditions of proteins, such as their concentrations, should be considered, and the prevalence of variations in a population may be useful for more reliable estimations of the functional impacts of mutations.

## Conclusion

In this study, we conducted a comprehensive analysis of SNVs at protein interfaces to clarify the relationship between the interfacial locations and the MAF of variants. We demonstrated that rare variants are more abundant in interfacial core regions when compared with common variants, as a result of evolutionary pressure, although certain exceptions exist, such as when the concentration of a protein is sufficient to prevent dissociation caused by a mutation at a critical binding site. In terms of amino acid substitutions, rare variants show that their randomness originated from nucleotide changes in DNA sequences, whereas common variants have bias probably due to the functional constraints of proteins. Our study sheds light on how the allele frequencies of variants are related to the protein functional sites. This information provides fundamental knowledge for further discussions of genetic variants of proteins and will lead to a deeper understanding of the potential deleteriousness of rare variants at the protein structural level.

## Materials and Methods

We started with 1,936,451 SNVs found in 6503 individuals by the NHLBI Exome Sequencing Project.^14^ After mapping onto the RefSeq^15^ sequences, 1,074,023 variants were located within protein coding regions and 638,105 were nonsynonymous substitutions. The variants in the coding regions were then mapped onto the 3D structures by searching the Protein Data Bank^21^ using BLAST.^16^ We used structures with more than 95% sequence identity with the query sequence. The structures were further checked by the PDBePISA^17^ server to eliminate monomers. Protein redundancy was removed by a sequence identity threshold of 50%. Finally, we obtained 20,305 variants on 1,343 protein complexes, and 4,204 variants were located on protein interfaces. The mapped SNVs were further divided into rare (MAF ≤ 1%), intermediate (1% < MAF ≤ 5%), and common (MAF > 5%). The interface residues on protein complexes were classified into three categories (core, rim, and support) based on the accessible surface area before and after binding, as proposed previously.^18^ The accessible surface areas of residues were calculated by NACCESS.^22^

## Supporting information

Supporting Information
