# Supplementary material for: Distribution of single‐nucleotide variants on protein–protein interaction sites and its relationship with minor allele frequency
Source: Protein Sci. 2015 Dec 9;25(2):316–21. doi: 10.1002/pro.2845 (PMC4815344; doi:10.1002/pro.2845)
Supplement: Supplementary file 1 — Supporting Information [file PRO-25-316-s001.doc]

**Supplementary Material**

**
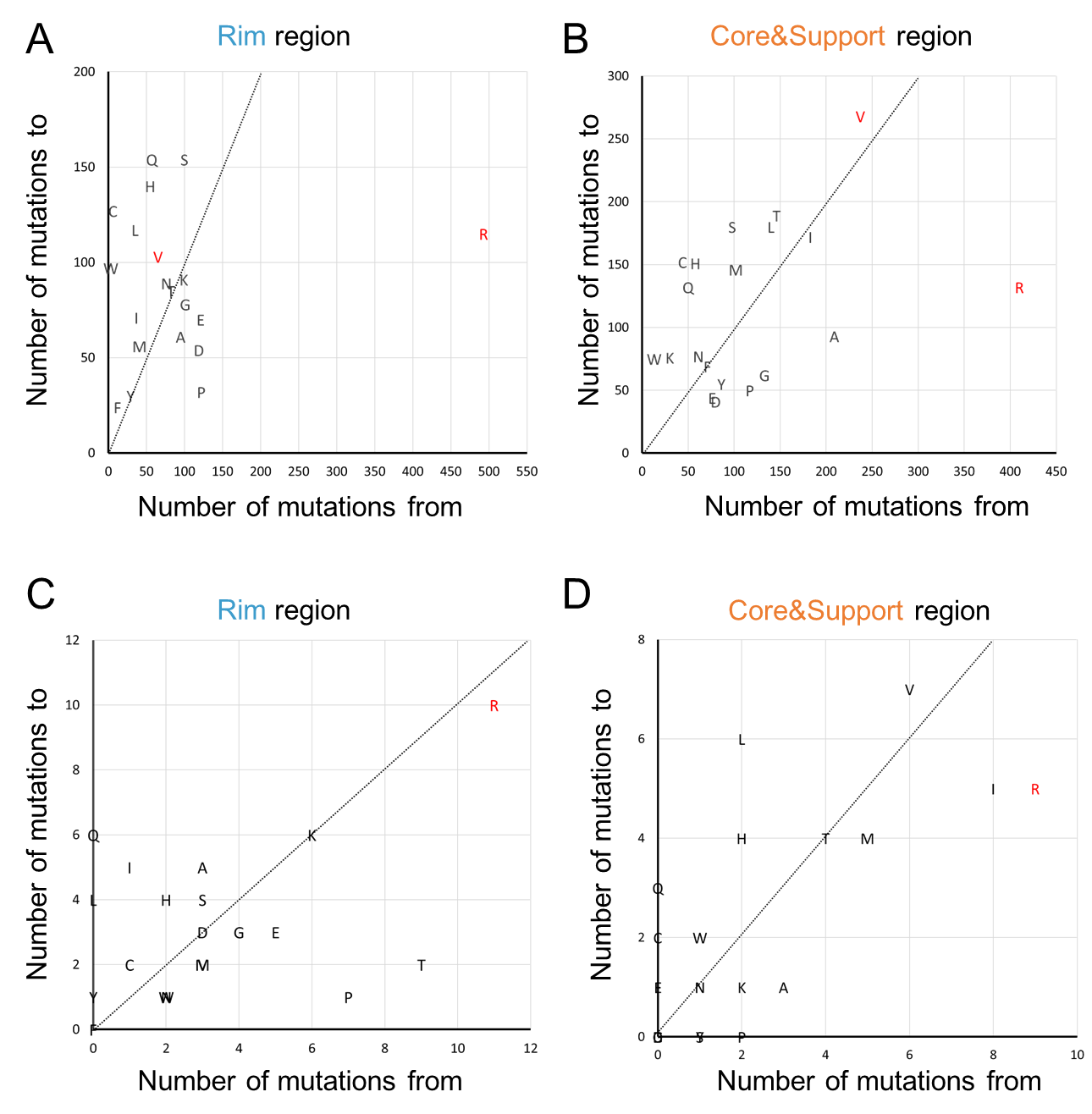
**

**Figure S1. Actual counts of amino acids of variants on protein interfaces.** Rare variants on the rim (A) and core & support (B) regions, and intermediate/common variants on the rim (C) and core & support (D) regions are shown. Here we combined the core and support regions into one category, due to the small numbers of mutations on these two regions.　Residues mentioned in the main text are highlighted in red. In (C), W and N, and M and V are overlapped at (2,1) and (3,2), respectively. In (D), E and F, G and D, and Y and S are overlapped at (1,0), (0,0), and (1,0), respectively.


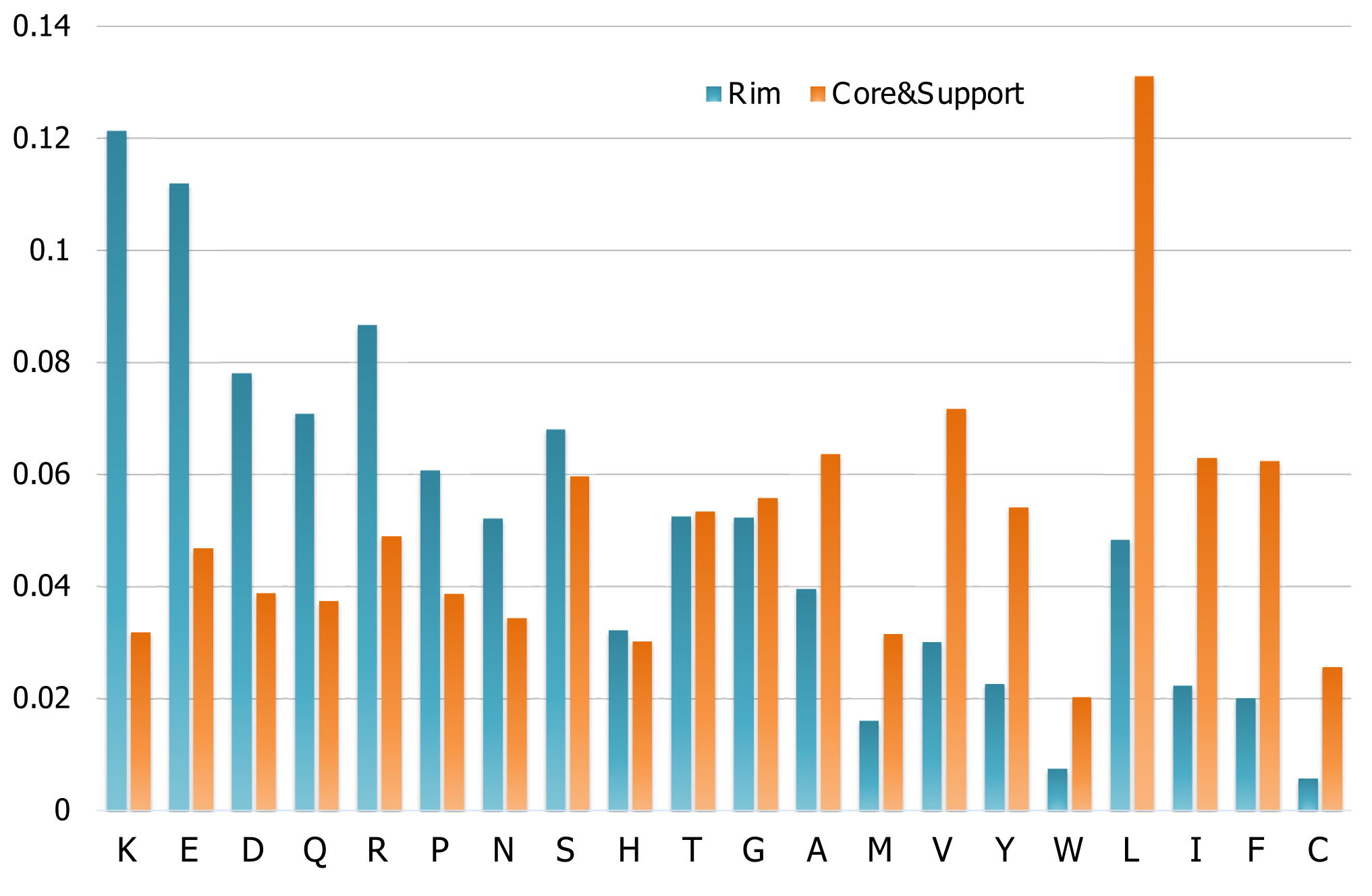


**Figure S2. Amino acid compositions of non-variant sites in different interfacial locations.** Amino acids are sorted by the ratio of rim to core/support regions.
